# Supplementary material for: BRAVE-NET: Fully Automated Arterial Brain Vessel Segmentation in Patients With Cerebrovascular Disease
Source: Front Artif Intell. 2020 Sep 25;3:552258. doi: 10.3389/frai.2020.552258 (PMC7861225; doi:10.3389/frai.2020.552258)
Supplement: Supplementary file 1 [file Data_Sheet_1.docx]

# Appendix I. - Model card

## Model Details

Developed by the Charite Lab for AI in Medicine (CLAIM) research group at Charite University Hospital, Berlin, main developer and person to contact: Adam Hilbert (adam.hilbert@charite.de)

Version: v1.0, 2020

Multiscale 3D Unet architecture, additional encoding path to capture contextual information with larger input patch down-sampled to half resolution. Dice Coefficient Score (DSC) loss was used to train the model. Deep supervision of decoder layers was applied by upsampling to output dimensions and generating output prediction to be added to DSC loss.

Hilbert et.al., 2020 “BRAVE-NET: High Performance Brain Vessel Segmentation In Patients with Cerebrovascular Disease”


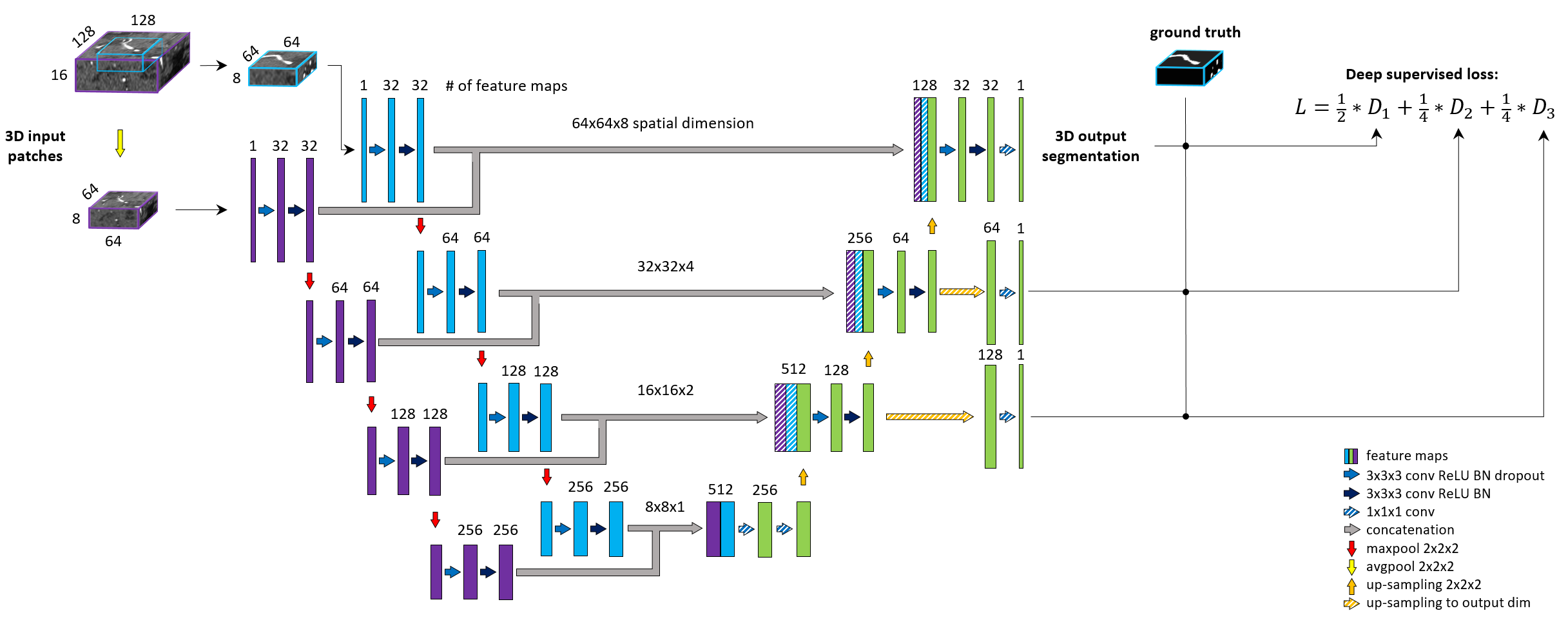


## Intended Use

Automatic brain vessel segmentation in MRI TOF images.

Accelerating and facilitating inspection of brain vessels in clinical practice for diagnosis of vessel abnormalities. Generating high quality segmentation input for other quantitative models to yield potential biomarkers of cerebrovascular status for diagnostic stratification.

The framework is general enough to be trained to segment other vessels or structures of similar nature as well as to be trained with other imaging modalities.

## Factors

Relevant factors that might affect performance include groups of patients with different cerebrovascular disease and abnormalities as well as artefacts, noise in imaging and different configurations of scanners. Furthermore, model convergence and performance highly depends on the quality of the presented ground truth segmentations.

Due to lack of data, influence of patient groups, pathologies and imaging factors was not evaluated.

## Metrics

Model was evaluated with five different metrics: Precision, Recall, DSC, 95 percentile Hausdorff Distance (95HD) and Average Hausdorff Distance (AVD). The evaluation metrics were calculated on whole brain segmentations (reconstructed from patch-wise predictions) to reflect the use-case, by using the open source evaluation tool from [(Taha and Hanbury, 2015)](https://www.zotero.org/google-docs/?jcsJZG).

Evaluation was done in a 4-fold cross-validation framework. 75% of all data was used for training and 25% for testing in each fold. Average performance and standard deviation across folds are reported.

## Training Data

Retrospective data of 264 patients with cerebrovascular disease from PEGASUS [(Martin et al., 2015)](https://www.zotero.org/google-docs/?7aWwjC), 7UP [(Ultrahigh-Field MRI in Human Ischemic Stroke – a 7 Tesla Study)](https://www.zotero.org/google-docs/?18pEYY) and 1000Plus [(Hotter et al., 2009)](https://www.zotero.org/google-docs/?DnNARN) studies was included in total.

No pre-/post-processing of data was done.

For PEGASUS and 7UP data, ground truth labels were generated by steps of: 1) pre-labeling with MeVisLab (<https://www.mevislab.de/>) region growing module 2) cross-checking by 2 junior raters 3) final check by experienced rater. For 1000Plus step 1) was replaced by a previous Unet segmentation model (Livne et al. 2019, v1.0).

2000 patches were extracted from each patient image for training, ensuring 50% of the samples were vessel-centric.

## Evaluation Data

Evaluation was done on whole brain segmentations using the automated tool from [(Taha and Hanbury, 2015)](https://www.zotero.org/google-docs/?oDLVsX). External validation was done on 20 healthy volunteers from the public MIDAS data collection, available at: <https://www.insight-journal.org/midas/community/view/21>. Labels were generated with the same procedure as for 1000Plus dataset and published at <http://doi.org/10.5281/zenodo.3968844>.

## Quantitative Analyses

Results shown in comparison of a standard Unet baseline. An asterisk (*) indicates significantly better performance of BRAVE-NET using the Wilcoxon Signed-Rank Test ($z_{score}>z_{critical}$ at 5% significance level).

| **Model** | **DSC (SD)** | **AVD (SD)** | **95HD (SD)** | **Precision (SD)** | **Recall (SD)** |
| --- | --- | --- | --- | --- | --- |
| **In-house data** | | | | | |
| Unet | 0.928 (0.004)* | 0.232 (0.041)* | 33.259 (1.060)* | 0.928 (0.005)* | **0.929 (0.004)** |
| BRAVE-NET | **0.931 (0.003)** | **0.165 (0.013)** | **29.153 (0.988)** | **0.941 (0.005)** | 0.923 (0.004) |
| **External data** | | | | | |
| Unet | 0.710 (0.038)* | 2.044 (0.473)* | 30.636 (7.157)* | 0.659 (0.049)* | 0.773 (0.044) |
| BRAVE-NET | **0.746 (0.036)** | **1.587 (0.461)** | **29.233 (7.027)** | **0.720 (0.049)** | **0.778 (0.055)** |

## Caveats and Recommendations

We recommend fine-tuning optimizer parameters for the specific dataset at hand, as well as experimenting with network parameters in sight of different image input (e.g. matching patch size to image resolution).

We note certain caveats in case of clinical application originating from the specific population of patients and pathologies present in the dataset. Our models were trained on MRA-TOF images of patients with chronic steno-occlusive disease (N=83) and acute ischemic stroke (N=181), all registered in Germany. Model performance might differ in case of:

- Patients with other cerebrovascular disease
- Patients of different ethnics
- Different vascular imaging modality (e.g. Computed Tomography Angiography or Digital Subtraction Angiography)
- In presence of further pathologies or neurological disease affecting cerebral vasculature (e.g. aneurysm, alzheimer disease, multiple sclerosis)

Furthermore, we note a limited performance in presence of rare pathologies such as cortical laminar necrosis.

# Appendix II. - Detailed results

| **Fold 1** | | | | | |
| --- | --- | --- | --- | --- | --- |
| **Models (all 3D)** | **DSC (SD)** | **AVD (SD)** | **95HD (SD)** | **Precision (SD)** | **Recall (SD)** |
| Unet | 0.933(0.026)* | 0.192(0.153)* | 32.938(9.711)* | 0.933(0.036)* | 0.934(0.03) |
| DS-Unet | 0.931(0.026)* | 0.207(0.165)* | 32.650(9.84)* | 0.928(0.034)* | 0.935(0.032) |
| Context-Unet | 0.935(0.025) | **0.145(0.082)** | **27.588(7.844)** | 0.936(0.033)* | **0.935(0.03)** |
| BRAVE-NET | **0.935(0.026)** | 0.156(0.095) | 30.498(7.536) | **0.948(0.029)** | 0.923(0.035) |
| **Fold 2** | | | | | |
| **Models (all 3D)** | **DSC (SD)** | **AVD (SD)** | **95HD (SD)** | **Precision (SD)** | **Recall (SD)** |
| Unet | 0.926(0.032)* | 0.282(0.286)* | 34.802(8.486)* | 0.930(0.037)* | 0.924(0.042) |
| DS-Unet | 0.928(0.029)* | 0.224(0.235)* | 34.647(9.768)* | 0.928(0.034)* | **0.928(0.04)** |
| Context-Unet | **0.932(0.029)** | 0.186(0.211) | **27.729(6.66)** | 0.941(0.033) | 0.925(0.038) |
| BRAVE-NET | 0.931(0.029) | **0.185(0.195)** | 29.183(7.474) | **0.942(0.031)** | 0.921(0.043) |
| **Fold 3** | | | | | |
| **Models (all 3D)** | **DSC (SD)** | **AVD (SD)** | **95HD (SD)** | **Precision (SD)** | **Recall (SD)** |
| Unet | 0.929(0.031)* | 0.248(0.388)* | 32.910(9.603)* | 0.929(0.045)* | 0.931(0.037) |
| DS-Unet | 0.929(0.029)* | 0.190(0.142)* | 33.339(9.669)* | 0.930(0.044)* | 0.930(0.032) |
| Context-Unet | 0.932(0.03) | 0.164(0.091) | 31.299(8.812)* | 0.927(0.046)* | **0.938(0.03)** |
| BRAVE-NET | **0.933(0.029)** | **0.158(0.08)** | **28.170(6.611)** | **0.937(0.037)** | 0.929(0.04) |
| **Fold 4** | | | | | |
| **Models (all 3D)** | **DSC (SD)** | **AVD (SD)** | **95HD (SD)** | **Precision (SD)** | **Recall (SD)** |
| Unet | 0.924(0.037)* | 0.208(0.11)* | 32.385(9.145)* | 0.921(0.045)* | 0.928(0.046) |
| DS-Unet | 0.922(0.037)* | 0.266(0.511)* | 33.044(9.632)* | 0.922(0.048)* | 0.923(0.049) |
| Context-Unet | 0.925(0.045) | 0.296(1.029) | 30.501(10.461) | **0.932(0.058)*** | 0.921(0.051) |
| BRAVE-NET | **0.927(0.037)** | **0.162(0.091)** | **28.760(8.038)** | 0.919(0.036) | **0.937(0.055)** |

Table 1. Test results of cross-validation for all models, average (SD) over all patients in each fold are given.  An asterisk (*) indicates significantly better performance of BRAVE-NET using the Wilcoxon Signed-Rank Test (z_score>z_critical at 5% significance level)

# Appendix III. – Mathematical formulations

## Encoder outputs

The output of level $l$ of the encoder path is computed as follows:

$$X_{e}^{l}=$$

$${Seq}_{2}\left( \left\{ \sum_{i=1}^{I_{l}} F_{i,m}^{2}*{Seq}_{1}\left( \left\{ \sum_{i=1}^{I_{\left( l-1 \right)}} F_{i,m}^{1}*M\left( X_{e, i}^{(l-1)} \right) \left| m\epsilon I_{l} \right. \right\} \right) \left| m\epsilon I_{l} \right. \right\} \right)$$

,where $I_{(l-1)}$ is the number of features maps in the output of the previous level (or 1 in case $l=1$), $X_{e,i}^{(l-1)}$ the $i$th feature map in the output of the previous level (or input patch in case $l=1$), $M\left( \cdot\right)$ the Max-pooling operation with 2x2x2 kernels (or identity function in case $l=1$), $F^{1}$ and $F^{2}$ the sets of the designated convolutional kernels for the 1^st^ and 2^nd^ convolution in the level, $(*)$ the convolutional operation, ${Seq}_{1}$ a sequence of ReLU activation, Batch-normalization and Dropout and ${Seq}_{2}$ a sequence of ReLU activation and Batch-normalization.

## Decoder inputs

The input of level $l$ of the decoder path is computed as follows:

$$X_{d}^{l}=Concat\left\{ \begin{matrix} X_{e}^{l} \\ U\left( X_{d}^{(l+1)} \right) if l<L \end{matrix} \right\}$$

,where $L$ is the number of levels of the Unet, $X_{d}^{(l+1)}$ the output of the previous decoder level, $U\left( \cdot\right)$ the up-sampling operation with 2x2x2 kernels, $X_{e}^{l}$ the corresponding encoder output for level $l$.

In case of Context-Unet and BRAVE-NET the decoder inputs change as follows:

$$X_{d}^{l}=Concat\left\{ \begin{matrix} X_{e1}^{l} \\ X_{e2}^{l} \\ U\left( X_{d}^{(l+1)} \right) if l<L \end{matrix} \right\}$$

, where $X_{e1}^{l}$ and $X_{e2}^{l}$ are the outputs from the context and standard encoder path.

Decoder inputs are processed by the same sequence of operations as shown above for encoder outputs, then propagated to the next decoder layer.
